# Identification of the Mouse T Cell ADP-Ribosylome Uncovers ARTC2.2 Mediated Regulation of CD73 by ADP-Ribosylation

**DOI:** 10.3389/fimmu.2021.703719

**Published:** 2021-08-24

**Authors:** Mario Leutert, Yinghui Duan, Riekje Winzer, Stephan Menzel, Eva Tolosa, Tim Magnus, Michael O. Hottiger, Friedrich Koch-Nolte, Björn Rissiek

**Affiliations:** ^1^Department of Molecular Mechanisms of Disease, University of Zurich, Zurich, Switzerland; ^2^Department of Genome Sciences, University of Washington, Seattle, WA, United States; ^3^Department of Neurology, University Medical Centre Hamburg-Eppendorf, Hamburg, Germany; ^4^Institute of Immunology, University Medical Centre Hamburg-Eppendorf, Hamburg, Germany; ^5^Mildred Scheel Cancer Career Center HaTriCS4, University Medical Center Hamburg-Eppendorf, Hamburg, Germany

**Keywords:** NAD, CD73, ADP-ribosylation, T cells, ARTC2.2

## Abstract

Mouse T cells express the ecto-ADP-ribosyltransferase ARTC2.2, which can transfer the ADP-ribose group of extracellular nicotinamide adenine dinucleotide (NAD^+^) to arginine residues of various cell surface proteins thereby influencing their function. Several targets of ARTC2.2, such as P2X7, CD8a and CD25 have been identified, however a comprehensive mouse T cell surface ADP-ribosylome analysis is currently missing. Using the Af1521 macrodomain-based enrichment of ADP-ribosylated peptides and mass spectrometry, we identified 93 ADP-ribsoylated peptides corresponding to 67 distinct T cell proteins, including known targets such as CD8a and CD25 but also previously unknown targets such as CD73. We evaluated the impact of ADP-ribosylation on the capability of CD73 to generate adenosine from adenosine monophosphate. Our results show that extracellular NAD^+^ reduces the enzymatic activity of CD73 HEK cells co-transfected with CD73/ARTC2.2. Importantly, NAD^+^ significantly reduced CD73 activity on WT CD8 T cells compared to ARTC2ko CD8 T cells or WT CD8 T cells treated with an ARTC2.2-blocking nanobody. Our study provides a comprehensive list of T cell membrane proteins that serve as targets for ADP-ribosylation by ARTC2.2 and whose function may be therefore affected by ADP-ribosylation.

## Introduction

Ecto-ADP-ribosyltransferases (ARTCs) are cell surface enzymes that utilize extracellular nicotinamide adenine dinuleotide (NAD^+^) to covalently attach the ADP-ribose group of NAD^+^ to arginine residues of various cell surface proteins under the release of nicotinamide (1, 2). The mouse ARTC family consist of six members: GPI-anchored ARTC1, ARTC2.1 and ARTC2.2, ARTC3, ARTC4 and the soluble ARTC5 (3). ARTC2.1 and ARTC2.2 are the ARTCs predominantly expressed by cells of the murine immune system (4). ARTC2.1 is highly expressed on the cell surface of innate immune cells such as macrophages and microglia (5) and to some extent on T cells (6). In contrast, ARTC2.2 is highly expressed on most T cell populations. Further, it is worth noting that the ARTC2.1 encoding gene, *Art2a*, is inactivated by a premature stop codon in the C57BL/6 (B6) mouse strain, whereas other strains such as Balb/c carry an intact *Art2a* gene (7). Therefore, in B6 mice, ecto-ARTC activity in the immune system is limited to the T cell compartment.

Results from ADP-ribosylation assays using ^32^P-NAD^+^ or etheno-NAD^+^ as substrate, revealed that ARTC2.2 ADP-ribosylates a broad spectrum of membrane proteins (8–11). So far, a limited number of ARTC2.2 targets have been characterized. Among them are cell surface receptors such as the interleukin 2 (IL-2) receptor alpha subunit (CD25) (12) and the alpha chain of CD8 (CD8a) (13) molecule, both chains of the integrin LFA1 (11) and the ATP-gated ion channel P2X7 (14).

The functional impact of ADP-ribosylation on the target protein has been extensively studied in case of P2X7. ADP-ribosylation of P2X7 mediates NAD^+^-induced cell death of T cells co-expressing ARTC2.2 and high levels of P2X7, such as regulatory T cells (Tregs), natural killer T cells, T follicular helper cells and tissue-resident memory T cells (14–19). Consistently, injection of NAD^+^ induces temporary depletion of Tregs, thereby favoring anti-tumor responses (15). Cells expressing both ARTC2.2 and P2X7 are particularly affected by NAD^+^ released during cell preparation procedures, i.e. isolation of T cells from spleen, resulting in extensive cell death in subsequent *in vitro* assays or upon adoptive cell transfer (20). Further, it has been shown that ADP-ribosylation of CD25 dampens IL-2 signalling by regulatory T cells, as the presence of NAD^+^ reduced STAT1 phosphorylation in response to IL-2 stimulation (12). ADP-ribosylation of CD8a inhibits binding to MHCI and ADP-ribosylation of LFA-1 inhibits homotypic binding to LFA1 on other cells (13, 21)

Apart from interference with target protein function, ADP-ribosylation can also affect the binding of monoclonal antibodies. For example, binding of clone 53-5.8 to CD8a is inhibited by ADP-ribosylation whereas clone H35-17.2 is unaffected (13). Similarly, ADP-ribosylation of P2X7 affects binding of clone Hano43, whereas clone Hano44 is unaffected (22).

The functional and technical consequences of ADP-ribosylation of cell surface proteins warrant proteomic investigation of the tissue- or cell-specific ADP-ribosylome. A comprehensive list of ADP-ribosylted target proteins opens the perspective to investigate the potential impact of this post-translational modification on the target protein function. For this, we recently developed a method combining Af1521 macrodomain-based enrichment of ADP-ribosylated peptides with mass spectrometry analyses to identify ADP-ribosylation sites across the proteome (23). Using this approach we previously generated ADP-ribosylomes of HeLa cells and mouse liver (23), mouse skeletal muscle and heart (24), mouse embryonic fibroblasts (25) and mouse microglia (26). The goal of this study was to subject mouse spleen T cells to a comprehensive ADP-ribsylome analyses in order to identify new targets of ARTC2.2-mediated cell surface protein ADP-ribosylation. From T cells incubated with NAD^+^, we identified 67 ADP-ribosylated target proteins, including 48 plasma membrane and 16 Golgi/ER proteins.

## Material and Methods

### Mice

C57BL/6 mice were used for all experiments. ARTC2ko mice (Art2b^tm1Fkn^, MGI#2388827) (27) were backcrossed onto the C57BL/6J background for at least 12 generations. All mice were bred at the animal facility of the University Medical Center (UKE). All experiments involving tissue derived from animals were performed with approval of the responsible regulatory committee (Hamburger Behörde für Gesundheit und Verbraucherschutz, Veterinärwesen/Lebensmittelsicherheit, ORG722, N18/006). All methods were performed in accordance with the relevant guidelines and regulations.

### Preparation of Immune Cells

Spleen and liver tissue were mashed through a cell strainer (50 mL falcon strainer, 70 µm, GBO) using a syringe piston. Additionally, liver leukocytes were purified by running a percoll gradient. Cells were resuspended in 5 mL 33% percoll/PBS in a 15 mL Falcon tube, and centrifuged at 1600 rpm, 12°C, for 20 min. The pellet was washed once in PBS (ThermoFisher). Single cell suspensions were kept in FACS buffer containing 1 mM EDTA (Sigma) and 0.1% bovine serum albumin (Sigma). Erythrocytes were lyzed using an ACK lysis buffer (155 mM NH_4_Cl, 10 mM KHCO_3_, 0.1 mM EDTA, pH 7.2). Peritoneal macrophages were harvested from the peritoneal cavity by lavage with 5 mL cold PBS + 1 mM EDTA. In order to prevent T cell surface ADP-ribosylation during cell preparation, some mice were i.v. injected with 30 µg of the ARTC2.2-blocking nanobody s+16a (28) 30 min prior to sacrificing.

### Antibodies and Flow Cytometry

The following monoclonal antibodies were used for flow cytometric analyses: anti-CD3e-PE (clone 17A2, Biolegend), anti-ARTC2.2-AF647 [clone A109, Prof. Koch-Nolte (29)], anti-CD73-PE (clone TY/11.8, Biolegend), anti-CD8a-FITC (clone 53-6.7, Biolegend), anti-CD11b-FITC (clone M1/70, Biolegend). For protein harvesting, CD3^+^ T cells from spleen and liver were isolated by fluorescence activated cell sorting (FACS) on a BD FACS Aria III.

### T Cell Protein Harvesting

FACS-sorted spleen T cells where subjected to *ex vivo* treatment with 50 µM NAD^+^ (Sigma) whereas a second preparation of spleen T cells and the liver T cells were left untreated in order to identify targets that were ADP-ribosylated during cell preparation (30). NAD^+^ was washed away after 15 min of incubation at 4°C and cells were subsequently treated with ARTC2.2-blocking nanobody s+16a for 15 min to avoid ADP-ribosylation of cell surface proteins by intracellular ADP-ribosyltransferases during lysis with denaturing RIPA buffer (Sigma).

### Proteomic Sample Preparation and ADP-Ribosylated Peptide Enrichment

For buffer exchange, protein reduction, alkylation, poly to mono-ADP-ribose reduction by PARG (Poly(ADP-Ribose) Glycohydrolase) and tryptic digestion a modified FASP (filter-aided sample preparation) protocol (31) was applied. For each sample 100-200 μg protein extracts were reduced in 1 mM DTT for 30 min and subsequently transferred to a 0.5 mL molecular weight cut off centrifugal filter unit (Microcon-30kDa Milipore, Sigma) and centrifuged until all buffer was passed through the filter. Samples were alkylated for 15 min using urea buffer containing 20 mM chloroacetamide and washed once with 100 μL urea buffer (8 M Urea, 0.1 M Tris-HCl pH 8) and once with 100 µL PARG buffer (50 mM Tris-HCL pH 8, 10 mM MgCl_2_, 250 µM DTT, 50 mM NaCl). 0.5 µg recombinant PARG enzyme (in-house) in 100 µL PARG buffer was added on to the filter and incubated for 1 h. Filter was subsequently washed with 100 µL 50 mM ammonium bicarbonate buffer. On filter digestion was performed in 100 µL 50 mM ammonium bicarbonate using 5 µg sequencing grade modified trypsin (Promega) at room temperature overnight.

ADP-ribosylated peptide enrichment was performed as previously described (23). The peptide mixture was diluted in PARG buffer (50 mM Tris–HCl, pH 8, 10 mM MgCl_2_, 250 μM DTT and 50 mM NaCl) and binding was carried out for 2 h at 4°C using the Af1521 macrodomain GST-fusion protein coupled to glutathione-Sepharose beads. Beads were washed three times with PARG buffer and bound peptides were eluted three times with 0.15% TFA. The resulting mixture was desalted using stage tips packed with C18 filters.

### Mass Spectrometry Data Acquisition

Samples were analyzed using an Orbitrap Q Exactive HF mass spectrometer (Thermo Fisher Scientific) coupled to a nano EasyLC 1000 (Thermo Fisher Scientific). Peptides were loaded onto a reverse-phase C18 (ReproSil-Pur 120 C18-AQ, 1.9 μm, Dr. Maisch GmbH) packed self-made column (75 μm × 150 mm) that was connected to an empty Picotip emitter (New Objective, Woburn, MA). Solvent compositions in buffers A and B were 0.1% formic acid in H2O and 0.1% formic acid in acetonitrile, respectively. Peptides were injected into the mass spectrometer at a flow rate of 300 nL/min and were separated using a 90 min gradient of 2% to 25% buffer B. The mass spectrometer was operated in data‐dependent acquisition mode and was set to acquire full MS scans from 300–1700 m/z at 60,000 resolution with an automated gain control (AGC) target value of 3 × 10^6^ or a maximum injection time of 110 ms. Charge state screening was enabled, and unassigned charge states and single charged precursors were excluded. The 12 most abundant ions on the full scan were selected for fragmentation using 2 m/z precursor isolation window and beam‐type collisional‐activation dissociation (HCD) with 28% normalized collision energy. MS/MS spectra were collected with AGC target value of 1 × 10^6^ or a maximum injection time of 240 ms. Fragmented precursors were dynamically excluded from selection for 20 s.

### Mass Spectrometry Data Analysis

MS and MS/MS spectra were converted to Mascot generic format (MGF) by use of Proteome Discoverer, v2.1 (Thermo Fisher Scientific). The MGFs were searched against the UniProtKB mouse database (taxonomy 10090, version 20160902), which included 24’905 Swiss-Prot, 34’616 TrEMBL entries, 59’783 reverse sequences, and 262 common contaminants. Mascot 2.5.1.3 (Matrix Science) was used for peptide sequence identification with previously described search settings (32). Enzyme specificity was set to trypsin, allowing up to four missed cleavages. The ADP-ribose variable modification was set to a mass shift of 541.0611, with scoring of the neutral losses equal to 347.0631 and 249.0862. The marker ions at m/z 428.0372, 348.0709, 250.0940, 136.0623 were ignored for scoring. S, R, T, K, E, D and Y residues were set as variable ADP-ribose acceptor sites. Carbamidomethylation was set as a fixed modification on C and oxidation as a variable modification on M. Peptides are considered correctly identified when a mascot score > 20 and an expectation value < 0.05 are obtained. ADP-ribosylation sites were considered correctly localized with a localization probability of > 70%.

### Bioinformatic Analyses

For protein network visualization and GO enrichment analyses cytoscape (33), STRING database (v. 11) (34) and the cytoscape string app (35) were used. For the network visualization only highest confidence interactions are shown (≥0.9) and proteins were clustered using the cytoscape string app.

### HEK Cell Transfection

Human embryonic kidney (HEK) 293T cells were transfected with a pCMVSport6.1 plasmid encoding mouse *Nt5e* (CD73) using jetPEI transfection reagent (Polysciences Europe). Transfected cells were FACS-isolated every 3 - 4 days for high CD73 expression in order to generate stably transfected HEK cells. The stably transfected CD73^+^ HEK cells were then co-transfected with pME plasmid encoding for *Art2b* (ARTC2.2) in order to evaluate the impact of ADP-ribosylation on CD73 enzymatic activity.

### AMP Degradation Assay

1 × 10^4^ HEK 293 T cells were incubated with 50 μM NAD^+^ on ice for 30 min. Cells were washed with FACS buffer twice (1410 rpm, 5 min, 4°C). Cells were resuspended in 100 μL FACS buffer, subsequently 100 μL AMP were added to a final concentration of 10 μM and incubated at room temperature for 40 min. Cells were spin down (1410 rpm, 5 min, 4°C) and 25 μL supernatant was transferred to a solid white plate. 25 μL AMP-Glo Reagent I were added per well, mixed and incubated at room temperature for 30 min. This was followed by addition of 50 μL AMP-Glo Detection Solution per well and incubation for 60 min at room temperature. Plate was read with a plate-reading luminometer.

### HPLC CD73 Enzymatic Activity Assay

To determine the AMPase activity by high performance liquid chromatography (HPLC), 0.2 × 10^6^ CD8^+^ T cells or peritoneal macrophages were incubated with 1 µM 1,N^6^-etheno-AMP (eAMP, Biolog) for 30 min at 37°C. After the incubation, cells were removed (450 × g, 5 min, 4°C) and all samples were passed through 10 kDa size exclusion filters (10,000 × g, 10 min, 4°C, Pall Corporation) and stored at -20°C until analyses. The analyses was performed on reversed-phase HPLC system (Agilent Technologies) with a 250 mm × 4.6 mm C8 Luna column (5 µm particle size, Phenomenex) as stationary phase. The mobile phase consisted of different compositions of HPLC buffer A (20 mM KH_2_PO_4_, pH 6.0) and B (50% buffer A, 50% methanol), and elution of the nucleotides from the column resulted from an increasing methanol content in the mobile phase [0.0 min (0.0% buffer B), 5.0 min (0.0% buffer B), 27.5 min (100.0% buffer B), 30.0 min (100.0% buffer B), 32.0 min (0.0% buffer B), 43.0 min (0.0% buffer B)]. The signals in both systems were detected by fluorescence detection (230 nm excitation wavelength, 410 nm emission wavelength). Different amounts of etheno-nucleotides (Biolog) were measured to quantify eAMP and the degradation product etheno-adenosine (eADO).

### Statistics and Software

For statistical analyses, GraphPad Prism 8 was used. Two groups were compared using the student’s t test. Multiple groups were compared using oneway ANOVA in combination with Dunnett’s multiple comparison test. Analysis of flow cytometric data was performed using FlowJo (Treestar). The structure model of mouse CD73 (Q61503) was analyzed using Pymol software.

## Results

### Identification of Potential ARTC2.2 ADP-Ribosylation Targets on T Cells

The aim of this study was to reveal potential ARTC2.2 ADP-ribosylation targets of mouse T cells. These proteins are most likely ADP-ribosylated on arginine residues facing the extracellular environment. Based on our previous studies (24, 26) we hypothesized that it is possible to detect ARTC2.2 mediated ADP-ribosylation by mass spectrometry even on proteins extracted from relatively small numbers of FACS-sorted T cells. We aimed to map ADP-ribosylated proteins of T cells isolated from mice under basal conditions [i.e. after encounter with endogenous extracellular NAD^+^ *in vivo* or during cell preparation (30)] and after *ex vivo* treatment with exogenous NAD^+^. For this we applied our established mass spectrometry-based strategy with modifications to make it applicable to low sample input (23, 26).

**Figure 1:**
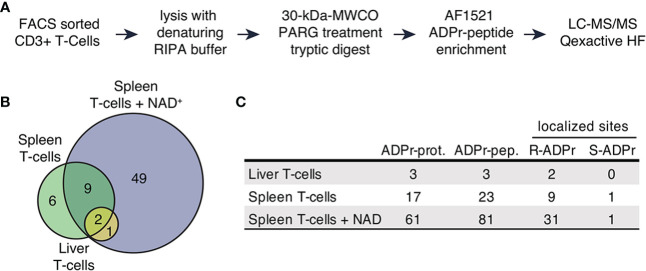
The ADP-ribosylated proteome identified in T cells. **(A)** Schematic workflow of proteomic sample processing, digestion and ADPr-peptide enrichment tailored to the low input protein amount obtained from FACS sorted T cells. **(B)** ADP-ribosylated proteins identified in the three different sample types depicted in a Venn diagram. **(C)** Numbers of uniquely identified ADP-ribosylated proteins, unique ADP-ribosylated peptides and modified amino acids that were confidently localized (localization probability > 70%). Modified arginine and serine sites were found.

CD3^+^ T cells were FACS sorted from seven spleens and livers of C57BL/6 mice. Aliquots of cells were incubated for 15min in the absence or presence of exogenous NAD^+^. To prevent ADP-ribosylation of intracellular proteins after cell lysis, cells were incubated with the ARTC2.2-blocking nanobody s+16a for 15 min before lysis with RIPA buffer. Proteins were subjected to filter-aided digestion and ADP-ribosylated peptides were enriched using the Af5121 macrodomain (**Figure 1A**). Samples were subsequently analyzed by mass spectrometry to identify peptides and to localize ADP-ribosyl modification sites. Importantly, we used higher-energy collisional dissociation (HCD) for peptide fragmentation, since this allows efficient identification of arginine ADP-ribosylated peptides due to the stability of ADP-ribosyl-arginine but is less effective in localizing serine and other O-linked ADP-ribosylations due to the lability of this modification type in HCD (24, 32, 36).

We identified 93 unique ADP-ribosylated peptides corresponding to 67 proteins (**Supplementary Table 1**). 49 ADP-ribosylated proteins were exclusively identified in T cells treated with NAD^+^ (**Figures 1B, C**), 12 ADP-ribosylated proteins were found in both untreated and NAD^+^ treated cells, 6 ADP-ribosylated proteins were only identified in the untreated conditions. We obtained confident ADP-ribose site localizations (localization probability >70%, considering R, S, T, Y, E, D, K as variable ADP-ribose amino acid acceptor sites) for 35 unique R- and 1 S-ADP-ribosylation sites (**Figure 1C**).

Taken together, our approach allowed us to identify a considerable number of ADP-ribosylated proteins from a low number of T cells. We observed induction of R-ADP-ribosylation upon treatment with exogenous NAD^+^ indicating active ARTC2.2 on these cells.

### Exogenous NAD^+^ Induces Extracellular ADP-Ribosylation of Proteins Relevant for the Immune Response

**Figure 2:**
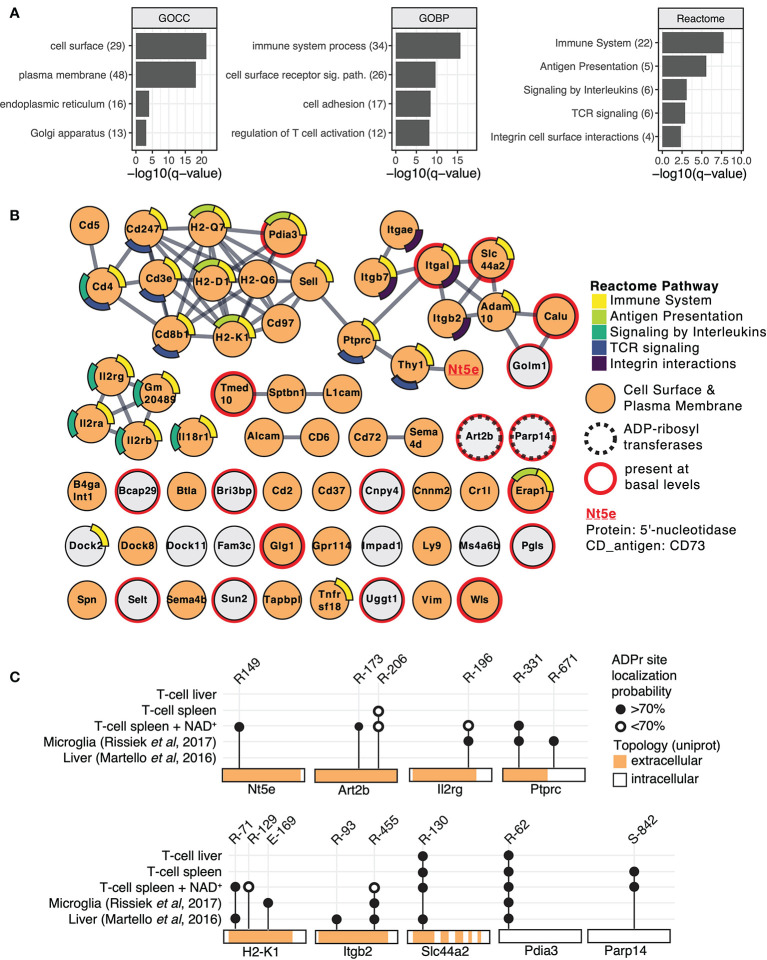
Mouse T cell surface ADP-ribosylome analyses. **(A)** Gene ontology term enrichment was performed for ADP-ribosylated proteins identified in all conditions against the whole mouse genome. Gene ontology biological processes (GOBP), gene ontology cellular components (GOCC) and Reactome pathways were included. The q-values of selected and significantly enriched terms are plotted. Numbers of ADP-ribosylated proteins included in the specific terms are indicated in brackets. **(B)** STRING protein-protein interaction network of ADP-ribosylated proteins identified in all conditions. Gene names of ADP-ribosylated proteins are shown and high confidence protein-protein interactions (STRING interaction score ≥0.9) are indicated with grey lines. Protein nodes are color coded by their affiliation to the Reactome pathways shown in **(A)** Proteins associated with the plasma membrane or cell surface are marked in orange. Proteins that were already identified under untreated conditions are marked with a red line and the two identified ADP-ribosyltransferases with a dashed line. CD73 that was chosen for follow up studies is highlighted. **(C)** ADP-ribosylation sites on selected proteins are plotted and compared to ADP-ribosylation sites identified in (23, 26).

To functionally cathegorize the identified ADP-ribosylation T cell target proteins, we performed gene-ontology (GO) term enrichment analysis, protein-protein interaction network visualization and literature comparisons. GO cellular component (GOCC) term enrichment analyses revealed strong enrichment for ADP-ribosylated proteins to be localized on the cell surface and plasma membrane (**Figure 2A**), providing further evidence that these proteins are most likely targets of ARTC2.2. Other significantly enriched cellular components were the endoplasmic reticulum and Golgi apparatus. GO biological processes (GOBP) were enriched in immune system processes, cell surface receptor signaling, cell adhesion and regulation of T cell activity (**Figure 2A**). Reactome pathway enrichment analysis provided additional separation of ADP-ribosylated proteins into more specific functional terms such as antigen presentation, signaling by interleukins, T cell receptor (TCR) signaling, and integrin cell surface interactions (**Figure 2A**).

Next, we performed protein level visualization of all identified ADP-ribosylated proteins by their association to Reactome pathway terms, relevant protein features, and protein-protein interactions. For this, we plotted the ADP-ribosylated proteins in form of a STRING network (**Figure 2B**) (34). ADP-ribosylated proteins with strong evidence for interactions among each other are connected with a grey line and proteins that had no interaction partners were preserved and shown as unconnected nodes. ADP-ribosylated proteins are color coded based on their affiliation to Reactome pathway terms identified in **Figure 2A**. ADP-ribosylated proteins are additionally marked if they are associated with the plasma membrane or cell surface (GOCC), are an ADP-ribosyltransferase or were already identified under basal conditions. The major hub of interacting proteins targeted by ADP-ribosylation was identified to have a role in TCR signaling, antigen presentation and cell surface integrin interactions. Connected to this cluster was *Nt5*e (5-prime-nucleotidase, CD73), a protein that hydrolyzes extracellular AMP to adenosine (37). An additional hub of interacting proteins consisted of the heteromeric IL-2 receptor complex, including IL2Rα, IL2Rβ and IL2Rγ that were all found to be ADP-ribosylated after addition of NAD^+^. We have previously identified IL2Rα as a target of ARTC2.2 and shown that its ADP-ribosylation functionally diminishes IL2 signaling (12). Most ADP-ribosylated proteins present at basal conditions were disconnected from these interaction hubs and less likely localized to the cell surface (**Figure 2B**). Two ADP-ribosyltransferases, ARTC2.2 (*Art2b*) and ARTD8/PARP14 (*Parp14*), were identified to be ADP-ribosylated, potentially by auto modification. Both were found to be ADP-ribosylated under basal and NAD^+^ treated conditions. Proteomic identification of ADP-ribosylation sites on ARTCs have previously been observed in mouse liver on ARTC2.2 (23), in mouse microglia cells on ARTC2.1 (26) and on ARTC1 in mouse heart and skeletal muscle tissues (24). ARTC (auto-)ADP-ribosylation can thus serve as a marker for ARTC activity. ADP-ribosylation by ARTD8/PARP14 has previously been associated with immune cell functions (38).

Next, we compared ADP-ribosylation levels of a few selected sites among the different conditions and with our previously published data on mouse liver (23) and microglia cells (26) (**Figure 2C**). ADP-ribosylation of CD73 at R149 was found exclusively on T cells treated with NAD^+^. Proteins that were identified in multiple different sample types showed modification on the same site (Pdia3, Slc44a2, Ptprc, Itgb2, Il2rg, H2-K1), or on additional sites (Ptprc, Itgb2, H2-K1) (**Figure 2C**). As exemplified by Pdia3 (a cytosolic protein exclusively modified on R62) and Slc44a2 (a multispan transmembrane protein exclusively modified on extracellular R130), R-ADP-ribosylation showed high site specificity on some proteins. In most of the analyzed cases, the R-ADP-ribosylation sites are located on the extracellular domain of the protein. An exception is the intracellular ARTD8/PARP14, which we found to be modified on S842 under basal and NAD^+^ treated conditions, consistent with our recent observation that ARTD8/PARP14 is modified by O-linked ADP-ribosylation (24). In summary, we identified numerous T cell surface proteins with immune system relevant functions that are R-ADP-ribosylated, likely by ARTC2.2, in the presence of exogenous NAD^+^.

### ADP-Ribosylation of CD73 Reduces the Capability of CD8 T Cells to Generate Adenosine

**Figure 3:**
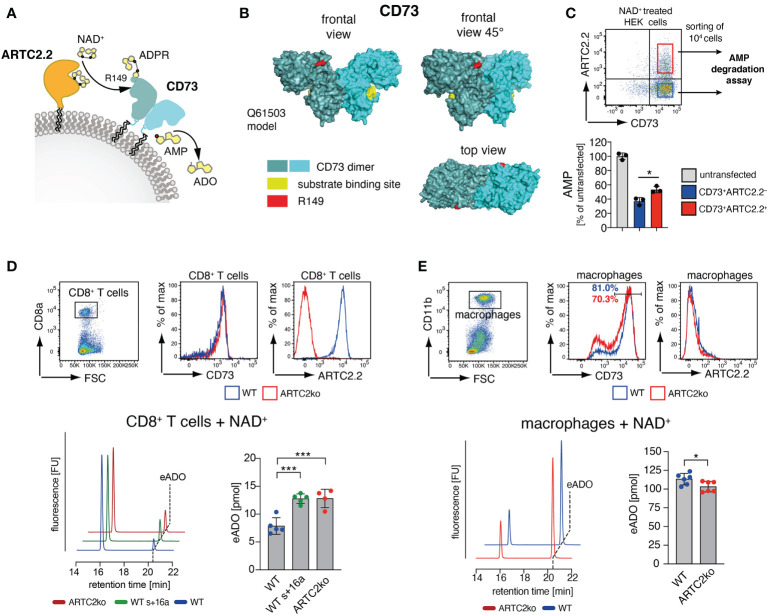
ARTC2.2-dependent regulation of CD73 enzymatic activity. **(A)** CD73 can degrade adenosine monophosphate (AMP) to adenosine ADO. ARTC2.2 ADP-ribosylates CD73 at R149, potentially interfering with enzymatic activity. **(B)** The structure model Q61503 of a mouse CD73 dimer is shown in cyan. ADP-ribosylation site R149 (red) and substrate binding site (yellow) are indicated. **(C)** ARTC2.2^+^CD73^+^ and ARTC2.2^–^CD73^+^ HEK cells (n = 3) were incubated with NAD^+^, FACS sorted and compared towards their capacity to degrade AMP in the AMPGlo assay. **(D)** Spleen CD8^+^ T cells from B6 WT, B6 WT treated with ARTC2.2-blocking nanobody s+16a, and from ARTC2ko mice were analyzed towards CD73 and ARCT2.2 expression. After NAD^+^ treatment, FACS sorted cells (n = 4-5 technical replicates) were further subjected to an HPLC-based assay to measure their capacity to generate etheno-ADO (eADO) from etheno-AMP (eAMP). **(E)** Peritoneal macrophages from B6 WT and ARTC2ko mice were analyzed towards CD73 and ARCT2.2 expression. After NAD^+^ treatment, FACS sorted cells (n = 6 technical replicates) were analyzed for their capacity to generate eADO. Statistical comparison of the two groups was performed by using the Student’s t test, comparison of more than two groups was performed by using oneway ANOVA analyses with Dunnett’s multiple comparisons (p < 0.05 = */p < 0.001 = ***). Data represent results from two **(C, E)** or three **(D)** independent experiments.

The majority of the identified T cell surface ADP-ribosylation targets are membrane proteins that act as receptors in cell signalling, antigen presentation or cell-cell adhesion. Apart from ARTC2.2 itself, CD73 was the only identified cell surface enzyme to be ADP-ribosylated. CD73 is expressed on several cell populations of the immune system, including regulatory T cells, CD8^+^ T cells and macrophages. It converts extracellular adenosine monophosphate (AMP) to adenosine (ADO) (**Figure 3A**), which acts as an immunosuppressant e.g. by inhibiting T cell proliferation (39). While ADP-ribosylation has been shown to impact the function of several cell surface receptors, little is known about the impact of ADP-ribosylation on the enzymatic activity of cell surface enzymes. Therefore, we investigated the impact of ADP-ribosylation on the catalytic activity of CD73. Analyses of the 3D structure model of mouse CD73 (Q61503) revealed that the identified ADP-ribosylation site R149 (red) is distant to the active site (yellow) of CD73 (**Figure 3B** **)**.

We first tested the impact of CD73 ADP-ribosylation in HEK cells stably transfected with mouse CD73 upon transient co-transfection with an expression vector for ARTC2.2. We pretreated these cells with NAD^+^ 24 h after transfection, and FACS-sorted equal amounts of CD73^+^ARTC2.2^–^ and CD73^+^ARTC2.2^+^ HEK cells (**Figure 3C**). The gates during cell collections were adjusted for equivalent cell surface levels of CD73 on ARTC2.2^–^ and ARTC2.2^+^ HEK cells. We then performed a comparative AMP degradation assay with the collected CD73^+^ARTC2.2^–^ and CD73^+^ARTC2.2^+^ HEK cells. We found that NAD^+^-treated CD73^+^ARTC2.2^+^ cells were slightly but significantly less potent in degrading AMP than NAD^+^-treated CD73^+^ARTC2.2^–^ HEK cells (**Figure 3C**), consistent with an inhibitory effect of ADP-ribosylation on CD73 activity.

We next analyzed the impact of NAD^+^ on the enzymatic activity of CD73 on primary T cells. For this we chose CD8^+^ T cells, which co-express ARTC2.2 and CD73, but are much less sensitive to NAD^+^-mediated cell death along the ARTC2.2/P2X7 axis when compared to Tregs (15). We isolated CD8^+^ T cells from untreated B6 WT mice, B6 WT mice treated with the ARTC2.2 blocking nanobody s+16a in order to block ARTC2.2 already *in vivo*, or from B6 ARTC2ko mice. Of note, cell surface levels of CD73 were comparable among WT and ARTC2.2ko CD8^+^ T cells (**Figure 3D**). Since CD8^+^ T cells express much lower cell surface levels of CD73 than CD73-transfected HEK cells, we used an HPLC-based assay measuring the CD73-dependent generation of etheno-adenosine (eADO) from etheno-adenosine monophosphate (eAMP), as this was a more sensitive approach compared to the AMP degradation assay. We treated all three samples with NAD^+^ and quantified the generation of eADO. Here, we observed that NAD^+^ treated WT CD8^+^ T cells generated less eADO compared to CD8^+^ T cells from s+16a treated WT mice or from ARTC2.2ko mice (**Figure 3D**). Finally, we performed a similar assay with peritoneal macrophages that express high surface level of CD73 but lack ARTC2.2 expression. Here, NAD^+^-treated peritoneal macrophages of WT mice generate slightly but significantly more eADO compared to ARTC2ko macrophages, consistent with the fact that a larger fraction of WT peritoneal macrophages expressed high level of CD73 when compared to ARTC2ko peritoneal macrophages (**Figure 3E**). In summary, we could demonstrate that in an NAD^+^-rich environment, CD73 enzymatic activity is dampened on cells co-expressing ARTC2.2.

## Discussion

In this study we investigated the T cell ADP-ribosylome with a focus on ARTC2.2-mediated ADP-ribosylation of T cell surface proteins. We identified 67 ADP-ribosylated target proteins 48 of which are expressed at the cell surface and 16 of which in the ER or the Golgi apparatus. Of the identified ADP-ribosylation sites 35 were on arginine residues and 1 on a serine residue. Many of the identified target proteins are involved in immune system processes such as signalling, cell adhesion and regulation of T cell activity. It is conceivably that ADP-ribosylation modifies the function of many of these target proteins, thereby fine tuning immune reactions (2, 20). As an example, we analyzed the impact of ADP-ribosylation on the capacity of CD73 to hydrolyze AMP into adenosine. Our results indicate that ADP-ribosylation of CD73 leads to a reduced conversion of AMP to adenosine.

In our study we analyzed the ADP-ribosylome of T cells treated with or without exogenous NAD^+^. It is important to note that NAD^+^ is also released during the preparation of murine T cells (30), which is sufficient to allow the ADP-ribosylation of T cell surface proteins. Therefore, we can not exclude that at least some of the observed ADP-ribosylated T cell surface proteins detected under basal conditions without addition of exogenous NAD^+^, such as *Slc44a2* or *Itgal*, identified in untreated T cells from spleen or liver were modified during cell isolation. To further investigate whether the observed ADP-ribosylation of proteins occurred during the cell isolation or is catalyzed *in vivo*, systemic injection of the ARTC2.2 blocking nanobody s+16a prior to cell harvesting would prevent ADP-ribosylation during preparation (17, 28). However, this would also block ADP-ribosylation *in vivo* during the time between injection and the sacrifice of mice, but would not prevent de-ADP-ribosylation by enzymes. In order to identify further targets that are ADP-ribosylated *in vivo* it might thus be necessary to block de-ADP-ribosylation. Finally, it would also be interesting to compare the ADP-ribosylome of different T cell types such as CD4^+^ helper T cells, CD4^+^ regulatory T cells or cytotoxic CD8^+^ T cells as well as the ADP-ribosylome of the same T cell population from different organs, e.g. spleen and liver. However, this would probably need large amounts of cell material to start with.

Our analyses of proteins derived from NAD^+^ treated T cells identified known and new targets of ARTC2.2-mediated ADP-ribosylation. We confirmed already known ARTC2.2 targets such as CD25 (*Il2rg*), CD8β (*Cd8b*) and CD45 (*Ptprc*). For CD25 we confirmed R196 (R178 in the mature protein) as ADP-ribosylation site, as reported in a previous study (12). Similarly, CD45 was found to be ADP-ribosylated on R331 of cells analyzed in the microglia study (26). Of note, the T cells used in this study were isolated from C57BL/6 (B6) mice, whereas the microglia study used cells from Balb/c mice. B6 mice carry a premature stop codon in the gene for the ARTC2.1 (40), and B6 T cells therefore exclusively express ARTC2.2 as cell surface ADP-ribosyltransferase (41). Balbc microglia express the thiol-activated ARTC2.1, but not ARTC2.2 and Balb/c T cells co-express ARTC2.1 and ARTC2.2 (5, 6). The finding of the same ADP-ribosylated targets on microglia and T cells indicates that these two closely related ADP-ribosyltransferases may share common targets and modify common sites.

MHC class I (MHC-I) molecules are yet not well characterized regarding the potential impact of ADP-ribosylation. In this study we identified H2-D, H2-K and the MHC-Ib molecule H2-Q to be ADP-ribosylated on T cells. MHC-I molecules present endogenous peptides to CD8^+^ T cells. Therefore, it would be interesting to test if MHC-I ADP-ribosylation affects its interaction with the T cell receptor (TCR) or loading of the peptide to form the MHC-I/peptide complex. Indeed, the identified R169 ADP-ribosylation site in H2-D1 is in close proximity to the TCR interaction surface (see PDB file 5m01). Further, a former study showed that ADP-ribosylation of the CD8β T cell coreceptor affects MHC-I/TCR interaction (13). It would thus be interesting to investigate, whether MHC-I ADP-ribosylation diminishes TCR binding in a similar fashion. Moreover, it is tempting to speculate that MHC-I ADP-ribosylation has an impact on TCR binding in an antigen-specific fashion: introduction of an ADP-ribose group at the MHC-I/TCR interaction site could lead to the activation of alternative CD8^+^ T cell clones that recognize this modified MHC-I/peptide complex. Future studies should address this hypothesis.

The list of ADP-ribosylation targets on T cells identified here is probably underestimated. The ATP gated P2X7 ion channel for example, a prominent and well characterized ARTC2.2 target on T cells (14), was not identified as target for ADP-ribosylation by our MS approach. P2X7 is expressed on regulatory T cells, NKT cells and CD4 effector/memory T cells (15, 17, 42) – together these cells constitute only a minor fraction of the T cells analyzed here. Therefore, the amount of available P2X7 proteins might have been below the detectable threshold. Furthermore, it is possible that ADP-ribosylated peptides are lost during sample preparation or mass spectrometry analysis due to technical circumstances.

In this study, we focused on the functional impact of CD73 ADP-ribosylation. CD73 is a ecto-5´-nucleotidase that generates immunosuppressive adenosine from AMP and thus plays a critical role in balancing the course of an inflammatory reaction (43). From a technical point of view, it is worth noting that adenosine is rapidly degraded to inosine by adenosine deaminase (ADA), both *in vivo* and *in vitro*. The etheno-adenosine (eADO) used in our HPLC-based assay to monitor and quantify CD73 enzymatic activity, however, is not a substrate for ADA (44) and therefore much more stable. Further, it has been recently shown that eADO is not taken up by cells *via* adenosine transporters (45). Therefore, differential degradation or uptake resulting in an experimental bias seems unlikely to explain the impact of NAD^+^ on the CD73 enzymatic activity. The identified ADP-ribosylation site at R149 is distant from the active site of CD73 and is therefore likely to act allosterically. When comparing CD8^+^ T cells and macrophages, NAD^+^ only had a dampening impact on the catalytic activity of CD73 on CD8^+^ T cells that co-express ARTC2.2 but not on macrophages that lack ARTC2.2. Therefore, CD73 ADP-ribosylation might be a T cell-specific mechanism that modulates CD73 activity in an NAD^+^ rich microenvironment, such as tumor tissue. Here, NAD^+^ could be released along with ATP during tumor cell secondary necrosis. Indeed, prostate cancer cell lines have been reported to actively release intracellular NAD^+^ into the culture medium (46). CD8^+^ T cells play a critical role in anti-tumor immune responses. CD73 on CD8 T cells seems to significantly contribute to the anti-tumor immunity response, since adoptively transferred CD73-deficient ovalbumin-specific OT-I T cells were more potent in killing OVA-expressing B16 melanoma tumors compared to WT OT-I T cells (47). This was accompanied by lower expression levels of the exhaustion markers programmed cell death protein 1 (PD-1) and CD39, strengthening the role of CD73 as an immune checkpoint and as a potential target in tumor therapy. Vice versa, it would be interesting to evaluate whether ARTC2-deficient OT-I T cells are less potent in killing OVA-expressing B16 melanoma, as CD73 activity would not be dampened by NAD^+^ in the tumor environment.

Interestingly, both ARTC2.2 and CD73 can be shed from T cells (48, 49) and it has recently been shown that soluble ARTC2.2 can ADP-ribosylate various cytokines, including IFNγ (50). Therefore, it would be interesting to elucidate whether soluble ARTC2.2 could also ADP-ribosylate the soluble form of CD73 and thereby control the cell-independent generation of adenosine.

## Data Availability Statement

The original contributions presented in the study are included in the article/**Supplementary Material**. Further inquiries can be directed to the corresponding authors.

## Ethics Statement

The animal study was reviewed and approved by Hamburger Behörde für Gesundheit und Verbraucherschutz, Veterinärwesen/Lebensmittelsicherheit.

## Author Contributions

BR and SM collected and prepared samples for mass spectrometry. ML performed mass spectrometry experiments, data collection and analyses. YD and RW performed CD73 functional assays. ET and TM assisted with CD73 functional assay data analyses and interpretation. FK-N and MH supervised the experiments and assisted with data interpretation and manuscript preparation. BR and ML assembled the figures and wrote the manuscript, which has been reviewed by all authors. All authors contributed to the article and approved the submitted version.

## Funding

This work was funded by the Deutsche Forschungsgemeinschaft (DFG, German Research Foundation) - Project-ID: 335447717 - SFB 1328 to FK-N (A10, Z2), TM (A13) and ET (A13/A14), a grant from “Hermann und Lilly Schilling-Stiftung für Medizinische Forschung” to TM, ADP-ribosylation research in the laboratory of MH is funded by the Kanton of Zurich and the Swiss National Science Foundation (grant 31003A_176177). ML is supported by a postdoctoral fellowship from the Swiss National Science Foundation (grant P400PB_194379). SM is supported by a grant of the Mildred Scheel Cancer Career Center HaTriCS4.

## Conflict of Interest

FK-N receives royalties from sales of antibodies developed in the lab *via* MediGate GmbH, a 100% subsidiary of the University Medical Center, Hamburg.

The remaining authors declare that the research was conducted in the absence of any commercial or financial relationships that could be construed as a potential conflict of interest.

## Publisher’s Note

All claims expressed in this article are solely those of the authors and do not necessarily represent those of their affiliated organizations, or those of the publisher, the editors and the reviewers. Any product that may be evaluated in this article, or claim that may be made by its manufacturer, is not guaranteed or endorsed by the publisher.
